# News media coverage of shisha in Nigeria from 2014 to 2018

**DOI:** 10.18332/tid/106139

**Published:** 2019-04-19

**Authors:** Emmanuel A. Abraham, Catherine O. Egbe, Olalekan A. Ayo-Yusuf

**Affiliations:** 1 Tobacco Control Unit, Non-Communicable Diseases Division, Federal Ministry of Health, Abuja, Nigeria; 2 Africa Centre for Tobacco Industry Monitoring and Policy Research, Sefako Makgatho Health Sciences University, Pretoria, South Africa; 3 Alcohol, Tobacco and Other Drug Research Unit, South African Medical Research Council, Pretoria, South Africa; 4 Faculty of Public Health, Sefako Makgatho Health Sciences University, Pretoria, South Africa

**Keywords:** Nigeria, shisha, tobacco, newspaper report

## Abstract

**INTRODUCTION:**

Shisha which was formally popular mostly in the Eastern Mediterranean region is now gaining popularity globally and has become a trending tobacco product in Nigeria. The increasing popularity of shisha is possibly driven by the attractive flavours and the misperception that it is safer than traditional cigarettes. Since the media plays a significant influence on public awareness, perception and understanding of various issues, this study sought to explore the coverage of shisha use by five national newspapers in Nigeria.

**METHODS:**

Five newspapers were selected based on their website traffic (online readership). The software NVIVO 12 was used to organise the content of the articles into themes.

**RESULTS:**

Between 2014 and 2018, we found 30 relevant publications about shisha. Some newspaper articles reported that shisha smoking was gaining popularity among youths, especially in tertiary institutions across the country. Similarly, some misconceptions about the safety of smoking shisha among shisha users was reported. Addiction to shisha was also reported as being so common that many shisha users were no longer satisfied with just visiting nightclubs and shisha cafes but now own the shisha paraphernalia. There were also reports that the tobacco in shisha was in some instances being replaced or mixed with other hard drugs like marijuana. Subtle advertisement of shisha lounges, which is a violation of the Nigeria National Tobacco Control Act, was also observed.

**CONCLUSIONS:**

There is a need for increased sensitization of the public through the media on the dangers of shisha smoking and other tobacco product use. There is also a need for a national survey to determine the prevalence of shisha use in Nigeria.

## INTRODUCTION

Shisha is known by different names depending on the region. It is also called waterpipe, hookah, narghile, or hubbly bubbly. It is a means of smoking tobacco in which the vapor passes through water before inhalation^1^. Tobacco is the main ingredient in shisha^2^, and tobacco use is the most important preventable cause of premature death and disease^3^. The World Health Organization (WHO) reports that every year, more than 7 million people die as a result of tobacco use globally^4^. Of this figure, more than 6 million are from direct tobacco use, and close to 0.9 million from exposure to secondhand smoke^4^. Low-income and middle-income countries like Nigeria bear nearly 80% of the global burden of tobacco-related mortality^4^.

According to WHO, shisha, which was invented in India by a physician known as Hakim AbulFath^5^, has been used to smoke tobacco and other substances by the indigenous people of Africa and Asia for at least four centuries^5^. But there is growing evidence to show that shisha is gaining popularity globally and is fast becoming a serious threat to public health^1^. A multi-country study conducted among school children 13–15 years old in several countries in the Arabian Peninsula (Bahrain, Oman, Qatar, United Arab Emirates, Kuwait and Yemen) showed that the prevalence of shisha smoking ranged from 9% to 15%^6^. Shisha smoking prevalence in this study was higher than cigarette smoking^6^.

In two cross-sectional studies conducted among medical students of two universities each in Pakistan and South Africa, the prevalence of shisha smoking was 21.5% and 18.6%, respectively^7,8^. Another cross-sectional study involving 427 university students in a private University in Kigali City, Rwanda, found the prevalence of ever smoking shisha to be 26.1%, and 20.8% for those that smoked in the last 30 days^9^. Another cross-sectional study conducted among 389 first-year students in a university in the Western Cape, South Africa, indicated that 40% of the respondents were current shisha users^10^. The mean age for first-time shisha smoking in this study was found to be 15.7 years^10^.

There is no published study on the prevalence of shisha smoking in Nigeria. However, data from the 2008 Global Youth Tobacco Survey (GYTS) conducted in 5 major cities in Nigeria showed the prevalence of reported current ‘other tobacco products’ use higher than cigarette smoking^11^. The consistently higher prevalence of other tobacco product use relative to cigarettes, in each of these cities, suggests that other tobacco products are becoming more popular than cigarettes^11^. One of these alternative tobacco products available in the Nigerian market is shisha. However, only limited information is available on the knowledge of health risks, attitudes and perception of Nigerians towards shisha smoking.

The media has a very significant influence on public awareness, normative believes, perception and understanding of various issues, and information relayed to the public through the media clearly influences health behavior^12^. For example, Friend and Levy^13^, in their review of several studies in the US, reported that mass media campaigns are associated with reduced smoking rates among adults and youths. In another study involving 98747 youths in the US, Smith et al.^14^ reported that each ten-article increase in newspaper volume over a five-month period was associated with, among others, increased odds of perceiving great harm from smoking, disapproving of smoking, and smoking in the past 30 days.

In Australia, newspaper coverage of tobacco issues has assisted tobacco control^15^. This could likely be one of the reasons why Australia is setting the pace globally in the implementation of tobacco control policies such as plain packaging for tobacco products.

Therefore, this study sought to explore the coverage of shisha use in Nigeria by qualitatively analyzing articles published in five of the most read online newspapers.

## METHODS

Major newspapers in Nigeria were ranked based on their website traffic^16^. The website traffic of newspapers was assessed on the *Alexa* website^16^. The five most popular newspapers based on their ranking, using this web tool, were then selected for the study. A similar study on newspaper coverage of the Ebola virus disease in Nigeria used four newspapers^17^. The five newspapers selected for this study according to their rankings include: Punch, Vanguard, Premium Times, The Nation, and The Guardian.

**Figure 1:**
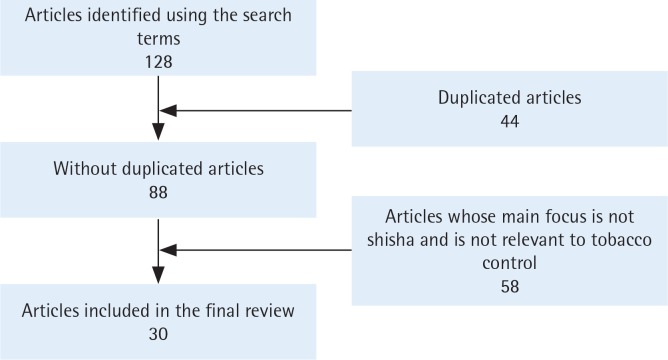
Flowchart showing selection of articles for analysis

The websites of each of these newspapers were searched between 12 to 28 July 2018 using the search terms: ‘shisha’, ‘Hookah’, and ‘water-pipe’. The inclusion criteria were: articles wholly or partly about shisha, hookah or water-pipe. Articles that did not address shisha smoking as a behavior or were not relevant to tobacco control were excluded in a second round of assessment. There were 30 articles (Figure 1) that met these inclusion and exclusion criteria. The first article was published on 13 July 2014 and the last one was published on 20 July 2018. Key information from each article was extracted and is shown in Table 1. The content of each newspaper article was extracted into a Word document individually and then exported into the software Nvivo 12. This software was used to organize the data into themes following a thematic analysis procedure by Braun and Clark^18^. This procedure includes: familiarising yourself with your data; generating initial codes; searching for themes; reviewing themes; defining and naming themes; producing the report^18^.

**Table 1:** Reports on Shisha in Nigerian newspapers 2014–2018

*S/N*	*Publication date*	*Code Name*	*Title of the media piece*	*Name of media*	*Journalist name (author)*	*URL/Links*	*Intended purpose of the media piece*	*Newspaper Section*
1	13-Jul-14	PT 1	Out in Abuja: Club behavior	Premium Times	Onyinye Muomah	https://www.premiumtimesng.com/entertainment/164861out-in-abuja-clubbehaviour.html	A report on a club in Abuja where ladies were openly smoking cigarettes and shisha	Entertainment
2	2-Oct-14	N 1	Students hooked on hookah	The Nation	Habeeb Whyte	http://thenationonlineng.net/students-hooked-onhookah/	To highlight the growing trend of shisha smoking among university students in Nigeria	Education (Campus Life)
3	7-Sep-15	V 1	I can smoke Shisha all day, every day, says Ella Mensah	Vanguard	Ayo Onikoyi	https://www.vanguardngr.com/2015/09/i-can-smoke-shisha-all-day-everyday-says-ella-mensah/	An actress's shisha smoking behaviour	News
4	20-May-16	G1	Uganda bans smoking in public places	The Guardian	N.A.	http://guardian.ng/news/uganda-bans-smoking-in-public-places/	A report on the new law in Uganda that bans the sale of electronic cigarettes and flavoured tobacco for water pipes or shisha	World (Africa)
5	17-Nov-16	V2	5 Spots to meet the one in Victoria Island (V.I.)	Vanguard	N.A.	https://www.vanguardngr.com/2016/11/5-spot-smeet-one-victoriaisland-v/	An advert of a restaurant and its services which includes ‘nice shisha’	News
6	24-Mar-17	G2	GL (Guardian Life) Lust List	The Guardian	N.A.	http://guardian.ng/life/food/gl-lust-list/	An advert on the various services of a restaurant including a shisha bar	Guardian Life (Food)
7	31-Mar-17	G3	Restaurant Review: La Veranda Is Ideal For Date Night	The Guardian	N.A.	http://guardian.ng/life/food/date-night-at-la-veranda/	To advertise the services of a restaurant which include ‘great shisha’	Guardian Life (Food)
8	8-Jun-17	G4	‘Tobacco industry frustrating enforcement of appropriate laws in Nigeria’	The Guardian	Edu Abade	http://guardian.ng/interview/tobacco-industry-frustrating-enforcement-of-appropriate-laws-innigeria/	To show that the Tobacco Industry is frustrating effective tobacco control in Nigeria; and to also show that flavoured cigarettes and shisha are not less harmful products	Interview
9	22-Jul-17	N2	The SHISHA pandemic	The Nation	Olatunji Ololade	http://thenationonlineng.net/the-shisha-pandemic/	An investigative report on the life of shisha smokers, reasons for their addiction, dangers they face, and the role of government in addressing this menace	Saturday Magazine Special Report
10	25-Aug-17	G5	Is Smoking Hookah Really Worse Than Cigarettes	The Guardian	Stella Ibru	http://guardian.ng/life/wellness/is-smoking-hookah-really-worse-than-cigarettes/	To show that shisha is not a safer form of tobacco smoking; Highlights the dangers of shisha use	Guardian Life (Wellness)
11	12-Sep-17	G6	Review: Backyard Grill In Victoria Island	The Guardian	Vanessa Walters	http://guardian.ng/life/food/review-backyard-grill-in-victoria-island/	To advertise what a restaurant offers which includes shisha	Guardian Life (Food)
12	22-Oct-17	G7	Review: A Slice Of Paris In Lagos Art Café	The Guardian	Beatrice Porbeni	http://guardian.ng/life/food/review-a-slice-of-paris-in-lagos-art-cafe/	To advertise the services of a restaurant which includes shisha	Guardian Life (Food)
13	7-Nov-17	G8	Big tobacco’s tiny targets and Africa’s challenge	The Guardian	Edu Abade	http://guardian.ng/features/focus/big-tobaccos-tiny-targets-and-africas-challenge/	To report a research by the Nigerian Tobacco Control Research Group (NTCRG) that found out that tobacco companies where targeting children by selling cigarettes, shisha and other flavoured products close to schools	Features (Focus)
14	15-Dec-17	G9	Rwanda bans water-pipe tobacco smoking, importation	The Guardian	NAN	http://guardian.ng/news/rwanda-bans-water-pipe-tobacco-smoking-importation/	To report the ban on use, advertisement and importation of water-pipe (shisha) tobacco in Rwandan	World (Africa)
15	15-Dec-17	P1	Rwanda bans water-pipe tobacco smoking, importation	Punch	(Xinhua/ NAN)	http://punchng.com/rwanda-bans-waterpipe-tobacco-smokingimportation/	To report the ban on use, advertisement and importation of water-pipe (shisha) tobacco in Rwandan	News
16	22-Dec-17	P2	Drug abuse worries Reps, want codeine, tramadol banned	Punch	John Ameh	http://punchng.com/drug-abuse-worries-reps-want-codeine-tramadol-banned/	Lawmakers worried about the increased abuse of drugs like shisha and its link to increase in domestic violence	News
17	12-Jan-18	V3	Sheraton launches BBQ night	Vanguard		https://www.vanguardngr.com/2018/01/sheraton-launches-bbq-night/	An advert of the services of a hotel which includes ‘Shisha with various flavours’	News, Travel & Tourism
18	16-Jan-18	G10	ERA/FoEN charges FG to emulate other African nations on shisha ban	The Guardian	Edu Abade	http://guardian.ng/news/era-foen-charges-fg-to-emulate-other-african-nations-on-shisha-ban/	To advocate for the ban of shisha as some other African Countries have done	Nigeria (National)
19	23-Jan-18	G11	Activists seek speedy enforcement of tobacco control act	The Guardian	Edu Abade	http://guardian.ng/news/activists-seek-speedy-enforcement-of-tobacco-control-act/	Advocate for enforcement of the nine key provisions of the NTC Act that do not require regulations; The need to commence mass public education; and a call for Nigeria to emulate some African countries by banning shisha	Nigeria (Metro)
20	26-Jan-18	N3	Group seek enforcement of tobacco control Act	The Nation	Omolara Akintoye	http://thenationonlineng.net/group-seek-enforcement-tobacco-control-act/	National Tobacco Control Alliance (NTCA) has urged the Federal Government to urgently enforce the National Tobacco Control (NTC) Act. A call for Nigeria to imitate Kenya, Tanzania and Rwanda in banning shisha use	News Update
21	10-Feb-18	G12	Olamide’s insult called ‘Science Students’	The Guardian	Gbenga Adebambo	http://guardian.ng/saturday-magazine/olamides-insult-called-science-students/	To report a trending song where the musician mentioned the use of illicit substances like Shisha etc., referring to those who use and mix them as science students	Youth Magazine
22	18-Mar-18	G13	Smoking Shisha Is Worse Than Smoking Cigarettes	The Guardian	Njideka Agbo	http://guardian.ng/life/smoking-shisha-is-worse-than-smoking-cigarettes/	To show that shisha smoking is worse than cigarette smoking, and to highlight the several constituents of shisha that makes it harmful	Guardian Life (Wellness)
23	30-Mar-18	N4	Stay away from Shisha pipes Russia warn players	The Nation	Julius Okorie	http://thenationonlineng.net/stay-away-shisha-pipes-russia-warn-players/	Russian Football Union (RFU) warned players against smoking Shisha pipes to avoid being linked to doping scandal. Especially as an Algerian footballer tested positive for cocaine after taking shisha	News Update, Sports
24	2-Apr-18	G14	Tackling big tobacco’s menace towards a smoke-free generation	The Guardian	Edu Abade	http://guardian.ng/features/focus/tackling-big-tobaccos-menace-towards-a-smoke-free-generation/	To report the statement by Michael R. Bloomberg at the WCOTH where he said more needs to be done to educate people about the dangers of tobacco uses like smoking, e-cigarettes, shisha and others	Features (Focus)
25	10-May-18	N5	Tackling drug abuse beyond codeine ban	The Nation	Kofoworola Belo-Osagie, Adegunle Olugbamila and Jane Chijioke	http://thenationonlineng.net/tackling-drug-abuse-beyond-codeine-ban/	A report after the FG banned cough syrup with codeine, where they advocated that Government should get to the root cause of addiction as banning is not the solution to codeine use and addiction because there were so many substances like shisha that youth are addicted to	Education
26	25-May-18	G15	ERA/FoEN predicts eight million deaths from tobacco smoking in 2020	The Guardian	Adamu Abuh	http://guardian.ng/news/era-foen-predicts-eight-million-deaths-from-tobacco-smoking-in-2020/	A report on an awareness campaign highlighting the dangers of tobacco and also pointing out that shisha in not a safer tobacco product	Nigeria (National)
27	5-Jun-18	P3	Enforce ban on Shisha, FG tells police, others	Punch	Eniola AKinkuotu	http://punchng.com/enforce-ban-on-shisha-fg-tells-police-others/	To enforce the ban on tobacco products with characterizing flavour which includes shisha	Featured
28	5-Jun-18	G16	Nigerians consume 20 billion cigarettes yearly, FG insists	The Guardian	Nkechi Onyedika-Ugoeze	http://guardian.ng/news/nigerians-consume-20-billion-cigarettes-yearly-fg-insists/	A press briefing by the Honourable Minster of Health were he reiterated the ban on tobacco products with characterizing flavours which includes Shisha because it has flavour	Nigeria (National)
29	6-Jun-18	PT 2	Nigerians consume 20 billion sticks of cigarette annually – Minister	Premium Times	Nike Adebowale	https://www.premiumtimesng.com/news/top-news/271095-nigerians-consume-20-billion-sticks-of-cigarette-annually-minister.html	To raise awareness on the danger of tobacco use	News (Top news)
30	6-Jun-18	P4	FG will continue to increase tax on tobacco	Punch	Eniola Akinkuotu	http://punchng.com/fg-will-continue-to-increase-tax-on-tobacco-minister/	To report the call made by the Minister of Health for security agencies to enforce the ban on flavoured cigarette including shisha	Health

## RESULTS

**Figure 2:**
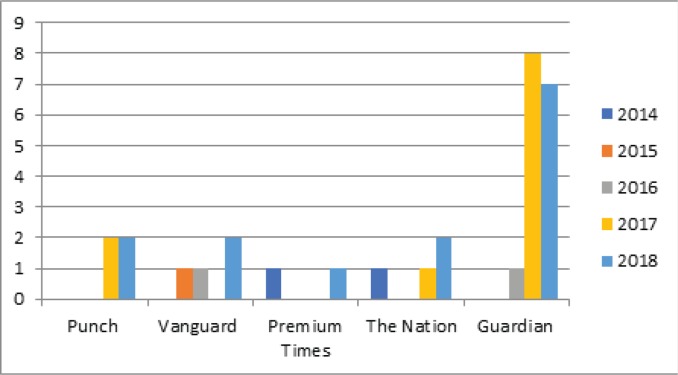
Newspaper coverage of shisha (2014–2018)

We found thirty relevant articles on shisha in the five newspapers searched. Figure 2 shows the number of newspaper reports on shisha from 2014 to 2018 for each of the newspapers used in this study. The following themes were developed from the articles: 1) Perception of prevalence of shisha smoking; 2) General misconception about safety of smoking Shisha; 3) Content of the shisha pot; 4) Addiction to shisha smoking; 5) Reasons for smoking shisha; 6) Financial cost of smoking shisha; 7) Health effects of shisha use; and 8) Laws regulating shisha use and its promotion.

### Perception of prevalence of shisha smoking

Six of the newspaper articles reported that shisha smoking was now a trend among youths especially in campuses of tertiary institutions across the country^19-24^. Shisha was reported as being popular among males and females and was being smoked by minors. The Nation newspaper, in an article dated 2 October 2014, reported that: *‘It is now everywhere on campuses today. We even have hookah competition, where students in various schools post their pictures on Facebook, Instagram and Twitter to compare thickness of smoke’*^24^.

### General misconception about safety of smoking shisha

Most shisha users perceived it to be enjoyable and a safer tobacco product because of the flavors and the fact that the tobacco smoke was passed through water. The Guardian, in an article dated 25 August 2017, reported on shisha smokers’ perception about the effect of smoking shisha: *‘…[they] see smoking shisha as a pleasant and relaxing experience. Approximately 44% of them believe shisha is not as harmful as cigarettes’*^22^. Several other newspaper articles also reported on this misconception about the safety of smoking shisha.

### Content of the shisha pot

There were reports indicating that the content of the shisha pot is usually not just tobacco^24-27^. The Nation newspaper, on 2 October 2014, reported: ‘*Normally, the bottom container is meant to be filled with water but students now fill it with gin and rum. Also, the flavour is meant to be inhaled without being mixed, but smokers here have devised means of mixing it with weed [marijuana] and other hard drugs’*^24^.

### Addiction to shisha smoking

Addiction to shisha was also reported by some of the newspapers^25,28,29^. The Nation newspaper in its 22 July 2017 article reported this about shisha addiction: *‘So rampant is the addiction that users have graduated from visiting night clubs and shisha cafes to ownership of shisha paraphernalia in their homes’*^25^.

### Reasons for increasing popularity of shisha smoking

Some of the likely reasons for the increasing popularity of shisha in society, deduced from the newspaper articles, are: 1) peer influence^25^, 2) misconception that shisha is less harmful than traditional cigarettes^22,24,25^, and 3) negative influence from social media and celebrities^29,30^. The Vanguard newspaper reported a comment made about shisha by a popular movie star: *‘I love shisha so badly that if all I wanted I have them and I don’t have to work again, I can smoke shisha 24/7 all day every day’*^29^. Other likely reasons for the increase in the use of shisha include: proliferation of shisha venues, cafes and bars^19,25^, and subtle shisha advertisements in the food and lifestyle section of some newspapers^31-35^.

### Financial cost of smoking shisha

The Nation newspaper in its 22 July 2017 article reported: *‘a small pack of fruit-flavoured tobacco was sold at N300 [less than $1] or more, while the cost of smoking shisha at a bar ranges from N2000 ($5.6) to N50000 ($138.9) depending on the location and associative drugs used’*^25^.

The paper also reported that the price of one shisha pot ranges from N10000 ($28) to N75000 ($208). Two other newspapers (The Guardian and The Nation) reported a price range of N1000 ($2.7) to N3500 ($9.7) for smoking shisha^21,24^.

### Health effects of shisha use

One common fact about shisha use highlighted in the newspapers and analysed in this study was the World Health Organization’s report that smoking shisha for one hour is equivalent to smoking about 100 cigarettes^21-24,26,36^. The Guardian article of 25 August 2017 reports that shisha users: *‘can increase the risk of contracting diseases, such as tuberculosis, hepatitis, meningitis and other infectious diseases’*^22^. This risk of smoking shisha was also reported by The Guardian and The Nation^25,26^.

### Laws regulating shisha use

Uganda, Tanzania, Rwanda, Pakistan, Jordan, Singapore and Saudi Arabia are countries reported to have banned shisha smoking and shisha bars^26^. The Guardian newspaper reported the call by a Civil Society Organisation, called Environmental Right Action/Friends of the Earth Nigeria, urging: *‘...the Federal government to emulate countries that have banned the smoking of waterpipe tobacco otherwise known as shisha, due to its addictiveness and deadly effect on the health of users’*^26^.

## DISCUSSION

In this study, newspaper articles that reported on shisha prevalence noted that it was now a growing trend among urban youths especially among university students. This trend is not peculiar to Nigeria; several authors have also reported this trend^9^. In a study of a nationally representative sample in Gambia, West Africa, it was observed that shisha was becoming increasingly popular^37^. Another cross-sectional study among 503 shisha users in Kuala Lumpur and the Area of Klang Valley in Selangor State, Malaysia also observes this increasing trend^38^. The reported rising trend of shisha smoking especially among young people globally may be due to several factors, such as the glamorization of shisha use by the media, peer pressure, the quest to appear ‘trendy’, availability of attractive flavours, and the misconception that shisha is a safer tobacco product compared to cigarettes^38^. Several other studies also show the increasing popularity of shisha use among females^38,39^. Reports of an increasing popularity of shisha use among females might not be unconnected with the aforementioned reasons; especially, attractive shisha flavours like strawberry, apple, pineapple that mask the harsh effects associated with the smoking of conventional cigarettes^40^.

A disturbing report in this study is the prevalence of shisha use among minors. Although the Nigerian Tobacco Control (NTC) Act 2015 prohibits the sale of tobacco to and by minors^41^, enforcement of this provision has not taken full effect^42^. Access to shisha by minors, as found in this study, shows that there is a lot to be done in the area of awareness raising and strict enforcement of the NTC Act. In a cross-sectional study among 1000 high school students in an Indian city, Anand et al.^43^ observed that 85.5% of minors reported that they had been to a hookah lounge. These authors therefore called for the legality of shisha lounges to be reconsidered since minors had access to them^43^.

Reports of the tobacco in shisha being mixed with marijuana and other hard drugs, as well as the water at the bottom of the shisha pot being replaced with gin or rum are likely signs of increasing addiction. It is very likely that the conventional shisha can no longer intoxicate enough some users, therefore the need to explore other options that could make them ‘feel high’. A qualitative study among 31 male and female students in a private university in Nigeria showed that shisha laced with drugs and codeine was favoured by female students over alcohol. The reason for this mixture could be the search for new sources of ‘highs’^43^. An online based study conducted among 3447 students from 8 colleges in North Carolina, USA, showed that 45% of respondents had marijuana and 18% had hashish in their hookah^44^. These shisha and hard drugs mixtures indicate that shisha could likely be a gateway to the use of other illicit drugs.

For a country where the current minimum wage is N18000 ($50) monthly, and over 50 per cent of its population lives below the poverty line^45^, spending between N1000 ($2.7) to N50000 ($138.9) for smoking shisha shows that the cost of maintaining a shisha addiction in the Nigerian context is financially huge.

Newspaper articles in this study that discussed the health effects of shisha smoking referred to the WHO report which stated that shisha smokers may inhale as much tobacco smoke/nicotine during one session as a cigarette smoker would inhale consuming a hundred or more cigarettes^5^. There is, therefore, the need for studies in Nigeria that link shisha use and its health consequences. There is also the need for increased awareness on the dangers of smoking shisha and other tobacco products.

Nigeria ranks 6th in the Global Tuberculosis burden^46^; is hyperendemic for hepatitis B^47^; and is within the Sub-Saharan Africa meningitis belt, which has the greatest incidence of the disease in the world^48^. The sharing of shisha pipes as it is done in shisha cafes and lounges is a risk factor that can increase the prevalence of these infectious diseases in Nigeria.

The NTC Act empowers the Standard Organisation of Nigeria (SON) to prescribe Industrial standards for tobacco products^41^. Although the current SON Industrial Standards for Cigarette bans cigarettes with characterizing flavours including menthol, SON does not at present have an Industrial Standard for shisha. Shisha importation, sale and use is therefore ‘free for all’. This is perhaps one of the reasons for the reported increase in the prevalence of shisha use in the country.

Among the newspaper articles reviewed, eleven had relevant information needed to warn the public about shisha use and the dangers associated with it. However, there were six articles that advertised shisha cafes in hotels and restaurants. The advertisement of shisha cafes is a violation of the National Tobacco Control Act^41^, which bans advertisement, promotion and sponsorship of any tobacco product. This violation further calls for the need to implement and enforce the NTC Act urgently.

With proper sensitization, the Nigeria press can do more in passing the right information about tobacco use. This can in turn shape public perception about tobacco and its products and influence policies that have positive impact on tobacco control. Australian newspaper reports of the Marlene Sharp case showed how powerful the media can be in opening up national discourse on tobacco related issues^49^. Marlene Sharp was a non-smoking bar worker in Australia who was awarded damages for laryngeal cancer caused by secondhand smoking. Analysis of the newspaper reports about this case by Wakefield et al.^49^ showed that the most common frame (27% of articles) advanced the view about the need for legislation to protect employees from the adverse health effects of passive smoking. With the growing trend of shisha use in Nigeria, increased media reporting on the health effects of shisha use can play a key role in shaping public perception about the risks associated with its use; it can also influence policies that favour public health.

This study shows that there is a need for either strict regulation of the content of shisha by the Standard Organization of Nigeria or outright ban of shisha by the Federal Government, as done by Uganda, Tanzania, Rwanda, Pakistan, Jordan, Singapore, and Saudi Arabia. Furthermore, there is an indication that there might be a need to tighten the regulation and/ or enforcement of regulations in relation to banning any form of promotion of shisha.

### Limitations and strengths

The restriction of our study to the five most popular Nigerian newspapers may have limited the data collected in this study. Also, opinions in the media articles cannot be generalized because these opinions were from a few people who were not systematically selected.

This study brings to the fore the need for an aggressive nationwide mass media campaign to enlighten people about the health effects of using shisha as well as other tobacco products. The findings of this study are also pointers to the direction regulatory activities should focus on, including: content of the shisha pot, access to minors, and strict ban on all forms of advertisements of shisha.

## CONCLUSIONS

Although there were not many newspaper articles about shisha in the period under review, this study found an increase in shisha reports within this period from 2 relevant articles in 2014 to 14 articles in 2018. The media plays a vital role in influencing people’s perception, the media also has a wider reach than scientific journals, therefore it is recommended that aggressive sensitization of the public through the media about tobacco control and provisions in the NTC Act be carried out. This will ensure that good and up-to-date information about the dangers of tobacco use (including shisha smoking) and how to quit, are made available to the reading public.

## CONFLICTS OF INTEREST

The authors have completed and submitted the ICMJE Form for Disclosure of Potential Conflicts of Interest and none was reported.

## References

[1] Aslam HM, Saleem S, German S, Qureshi WA (2014). Harmful effects of shisha: literature review. Int Arch Med.

[2] Butt M (2014). Emerging trends of Shisha smoking in Pakistani youth. World Fam Med J.

[3] Shaik SS, Doshi D, Bandari SR, Madupu PR (2016). Tobacco use cessation and prevention–A review. J Clin Diagn Res.

[4] World Health Organization (WHO) Tobacco.

[5] World Health Organization (2015). Advisory note: Waterpipe tobacco smoking: health effects, research needs and recommended actions for regulators.

[6] Maziak W, Taleb ZB, Bahelah R, Islam F, Jaber R, Auf R, Salloum RG (2015). The global epidemiology of waterpipe smoking. Tob Control.

[7] Senkubuge F, Ayo-Yusuf OA, Louwagie GMC, Okuyemi KS (2012). Water Pipe and Smokeless Tobacco Use Among Medical Students in South Africa. Nicotine Tob Res.

[8] Zavery A, Qureshi F, Riaz A, Pervez F, Iqbal N, Khan JA (2017). Water pipe (shisha) use and legislation awareness against shisha smoking among medical students: a study from Karachi, Pakistan. J Community Health.

[9] Omotehinwa OJ, Japheths O, Damascene IJ, Habtu M (2018). Shisha use among students in a private university in Kigali city, Rwanda: prevalence and associated factors. BMC Public Health.

[10] Daniels KE, Roman NV (2013). A descriptive study of the perceptions and behaviors of waterpipe use by university students in the Western Cape, South Africa. Tob Induc Dis.

[11] Ekanem IOA (2008). Global Youth Tobacco Survey for Nigeria Report.

[12] McCombs ME, Shaw DL (1972). The agenda-setting function of mass media. Public Opin Q.

[13] Friend K, Levy DT (2002). Reductions in smoking prevalence and cigarette consumption associated with mass-media campaigns. Health Educ Res.

[14] Smith K, Wakefield M, Terry-McElrath Y, Chaloupka F, Flay B, Johnston L (2008). Relation between newspaper coverage of tobacco issues and smoking attitudes and behaviour among American teens. Tob Control.

[15] Durrant R, Wakefield M, McLeod K, Clegg-Smith K, Chapman S (2003). Tobacco in the news: an analysis of newspaper coverage of tobacco issues in Australia, 2001. Tob Control.

[16] Alexa Internet, Inc Find Website Traffic, Statistics, and Analytics.

[17] Smith S, Smith S (2016). Media coverage of the Ebola virus disease in four widely circulated Nigerian newspapers: lessons from Nigeria. Health Promot Perspect.

[18] Braun V, Clarke V (2006). Using thematic analysis in psychology. Qual Res Psychol.

[19] Abade E Big tobacco’s tiny targets and Africa’s challenge.

[20] Adebowale N Nigerians consume 20 billion sticks of cigarettes annually -Minister.

[21] Agbo N Smoking Shisha Is Worse Than Smoking Cigarettes.

[22] Ibru S Is Smoking Hookah Really Worse Than Cigarettes.

[23] Muomah O Out in Abuja: Club behaviour.

[24] Whyte H Students hooked on hookah.

[25] Ololade O The shisha pandemic.

[26] Abade E ERA/FoEN charges FG to emulate other African nations on shisha ban.

[27] Okorie J Stay away from Shisha pipes Russia warn players.

[28] News Agency for Nigeria (NAN) Drugs abuse: Behavioural scientist raises alarm over teenage initiation.

[29] Onikoyi A I can smoke Shisha all day, everyday, says Ella Mensah.

[30] Adebambo G Olamide’s insult called ‘Science Students’.

[31] Vanguard Media Limited 5 spots to meet the one in Victoria Island (V.I).

[32] Guardian Newspapers Restaurant Review: LA Veranda Is Ideal For Date Night.

[33] Vanguard Media Limited Sheraton launches BBQ night.

[34] Porbeni B Review: A Slice of Paris in Lagos Art Café.

[35] Walters V Review: Backyard Grill in Victoria Island.

[36] Abuja ΑΑ ERA/FoEN predicts eight million deaths from tobacco smoking in 2020.

[37] Jallow IK, Britton J, Langley T (2017). Prevalence and determinants of tobacco use among young people in The Gambia. BMJ Glob Health.

[38] Wong LP, Alias H, Aghamohammadi N, Aghazadeh S, Hoe VCW (2016). Shisha Smoking Practices, Use Reasons, Attitudes, Health Effects and Intentions to Quit among Shisha Smokers in Malaysia. Int J Environ Res Public Health.

[39] Akingbade RE, Emmannuel OT (2018). ‘Hanging out’, trends in substance use among youth in private tertiary institutions in Nigeria: A qualitative study. Int Soc Sci J.

[40] Afifi R, Khalil J, Fouad F, Hammal F, Jarallah Y, Abu Farhat H (2013). Social norms and attitudes linked to waterpipe use in the Eastern Mediterranean Region. Soc Sci Med.

[41] Federal Republic of Nigeria (2015). National Tobacco Control Act, 2015.

[42] Egbe CO, Bialous SA, Glantz S (2018). FCTC Implementation in Nigeria: Lessons for Low and Middle-Income Countries. Nicotine Tob Res.

[43] Anand NP, Vishal K, Anand NU, Sushma K, Nupur N (2013). Hookah use among high school children in an Indian city. J Indian Soc Pedod Prev Dent.

[44] Sutfin EL, Song EY, Reboussin BA, Wolfson M (2014). What are young adults smoking in their hookahs? A latent class analysis of substances smoked. Addict Behav.

[45] World Bank Poverty & Equity Brief: Sub Saharan Africa-Nigeria.

[46] World Health Organization (2018). Global Tuberculosis Report 2018.

[47] Ikobah J, Okpara H, Elemi I, Ogarepe Y, Udoh E, Ekanem E (2016). The prevalence of hepatitis B virus infection in Nigerian children prior to vaccine introduction into the National Programme on Immunization schedule. Pan Afr Med J.

[48] Jafri RZ, Ali A, Messonnier NE, Tevi-Benissan C, Durrheim D, Eskola J (2013). Global epidemiology of invasive meningococcal disease. Popul Health Metr.

[49] Wakefield M, Smith KC, Chapman S (2005). Framing of Australian newspaper coverage of a secondhand smoke injury claim: Lessons for media advocacy. Critical Public Health.

